# The decline in the physical stature of the U.S. population parallels the diminution in the rate of increase in life expectancy

**DOI:** 10.1016/j.ssmph.2025.101872

**Published:** 2025-10-14

**Authors:** John Komlos

**Affiliations:** Department of Economics, Ludwig-Maximilian University of Munich, Germany

## Abstract

The U.S. healthcare and food-provisioning systems have failed to create an environment in which the human biological organism can flourish. Consequently, key health outcomes, most notably life expectancy, have consistently lagged those of other high-income populations since the Reagan era, coinciding with the adoption of economic policies that increased inequality and precarity across the population. We estimate the trends in physical stature, another omnibus indicator of a population's biological well-being that reflects not only nutritional intake, inequality, and stress experienced by the population, but also the overall health environment—using a sample of 44,322 adults from the NHANES surveys, stratified by gender and three ethnic groups. We find that the height of Americans began to decline among those born around or before the early 1980s in parallel with the diminution in the rate of increase of life expectancy. The decline in adult height ranged from 0·68 ± 0.36 cm among white women to 1·97 ± 0.50 cm among Hispanic men and is statistically significant across all six demographic groups considered. This decline in heights serves as corroborating evidence that the U.S.‘s laissez-faire approach to healthcare and food provisioning delivers suboptimal population health outcomes. Public health priorities urgently need to be refocused.

## Highlights

• The height of the U.S.-born population is analysed using 17 NHANES surveys.
• Declines ranged from 0.68±0.36 cm among white women to 1.97±0.50 cm among Hisp men.
• The inflection point occurred mostly by the late 1970s or early 1980s birth cohorts.
• The inflection point coincided with the decline in the rate of increase in life expectancy.

## 1 Introduction

**Fig. 1:**
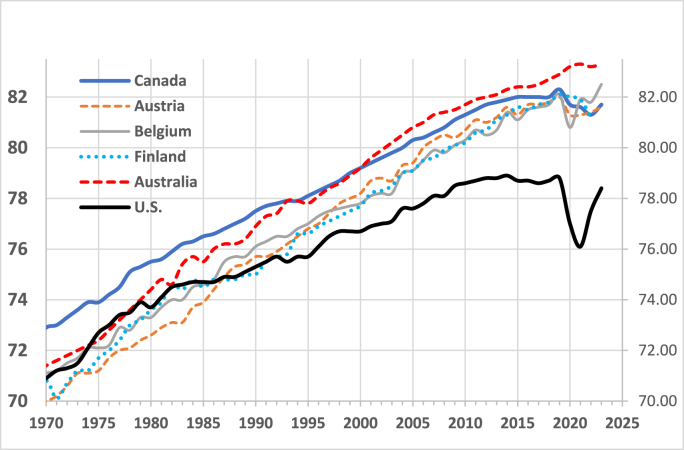
Life Expectancy Trends in Selected OECD Countries Source: OECD. Life expectancy at birth. OECD Data Explorer 2024; https://stats.oecd.org/#

Despite spending 17 % of its GDP on healthcare—twice the per-capita average of other wealthy countries— it is common knowledge that Americans are less healthy and live 3–4 years shorter lives than their counterparts in high-income countries (Fig. 1) (National Center for Health Statistics, 2020–2021; Banks et al., 2006). However, it is not widely recognized that U.S. life expectancy—an omnibus indicator of biological well-being—began to lag that of peer countries in 1983, coinciding with major ideological and policy shifts that prioritized market solutions to social problems over government provided social services and increased both inequality and the level of stress experienced by the population (Fig. 1) (Komlos, 2019; Thurow, 1985).

In 1983 the rate of increase in life expectancy began to decline. Between 1961 and 1982, life expectancy rose by an average of 0.21 years per year; however, from 1983 to 2009, this rate declined to 0.15 years, and between 2010 and 2019 it fell further to just 0.03 years per year.

This study examines another measure of public health—the physical stature of the U.S. population—which, like life expectancy, serves as a comprehensive indicator of the health status of children and youth (Bogin, 2020). The trends until the birth cohorts of 1986 were reported earlier (Komlos & Baur, 2004; Komlos & Lauderdale, 2007). The aim of this study is to extend the height estimates until the birth cohorts of 2003, who experienced their growing years until 2023.

## 2 Height is an omnibus indicator of biological well-being

Adult physical stature serves as a comprehensive indicator of how well the human organism thrives in its socio-economic environment during the first two decades of life (Komlos & Kelly, 2016; Tanner, 1987a). It is a “mirror” of society according to a popular metaphor (Tanner, 1987b), inasmuch as it captures the cumulative impact of a wide array of factors on the physical development of children and youth at the cellular level (Komlos et al., 2020; Steckel, 2008). Final adult height is affected by the accessibility and affordability of medical services, the cost of pharmaceuticals, the quality of perinatal and preventive health-care, the burden of disease, environmental pollution, psychological stress, family income, food prices, diet, water quality, inequality, poverty, social entitlements, and the strength of social safety nets—all of which together determine the extent to which individuals can experience healthy physical development (Bogin, 2001; Müller et al., 2022).

Attained height has health implications throughout the life course and correlates positively with practically all dimensions of the quality-of-life including longevity (Case et al., 2005; Jousilahti et al., 2000; NCD Risk Factor Collaboration (NCD-RisC), 2016; Benyi et al., 2019; Waaler, 1984). While both height and life expectancy serve as a proxy indicator of health, they differ in two important respects. First, height reflects conditions only during the first two decades of life, whereas life expectancy captures cumulative influences across the entire lifespan. Second, mortality is a binary measure, while height is a continuous one, allowing for a more nuanced assessment of biological development during the first twenty years of life until adulthood. These differences notwithstanding, most of the factors discussed here, inequality, stress, obesity, nutrition, and medical services, impact both variables. Thus, these indicators offer complementary perspectives on the overall effectiveness public health (Craig et al., 2016).

## 3 Data/method

Data used in this study are from the National Health and Nutrition Examination Surveys conducted by the Centers for Disease Control and Prevention through the National Center for Health Statistics. The surveys began in 1959, and continued through 18 waves until the most recent survey that ended in 2023 (National Center for Health Statistics, 2024). However, the first survey was not included in this study because strata and primary sampling unit identifiers were not reported separately. The number of observations used is 44,322 (Table 1). The physical examinations were conducted and recorded by professionals. We limit the analysis to individuals in three ethnic groups non-Hispanic whites, non-Hispanic blacks, and Hispanics between the ages of 20 and 50, when height remains constant. (Thereafter the label non-Hispanic is dropped. Asians are excluded because of small sample size.) Analysis was done using STATA/MP v.19.5 using sample weights with command svy: glm in order to account for the complex sample design.

**Table 1:** Number of observations, adults.

	Men	Women	Total
White	11,553	16,454	28,007
Black	4,916	6,573	11,489
Hispanics	2,152	2,674	4,826
Total	18,621	25,701	44,322

While the height of the U.S. population is widely reported, nearly all studies share two limitations that impede the understanding of its long-run development (Fryar et al., 2018). First, they focus on period effects (measurement years) while minimizing birth-cohort effects. However, an individual's adult height depends on factors that have accumulated between birth and age 20, the approximate time of reaching adult final height. After all, individuals within a birth cohort are typically exposed to comparable social, cultural, medical, technological, and economic factors throughout their life course, which collectively influence their developmental outcomes. This is clearly not true for measurement cohorts. Focusing on period effects makes it more difficult to understand the influence of nutritional circumstances, stress, inequality, medical care, and government provisioning of social services on physical stature. In contrast, for everyone born in 1960 faced a similar socio-economic environment that influenced their physical development. The distinction between birth-cohort and period effects is critical, as the environment begins shaping individual characteristics in utero and continues to do so from birth onward, leaving a lasting imprint that influences physical trajectories in a path-dependent manner (Almond & Currie, 2011; Barker, 1995). Hence, we analyze the data by birth cohorts in order to keep the nutritional and medical experiences homogeneous. To refrain from being excessively repetitive, unless otherwise noted, a reference to a date refers to the birth cohorts of that date.

The second major limitation of most studies is that they conflate individuals born in the U.S. with immigrants. This adds uncontrolled heterogeneity into the analysis, because immigrants’ food consumption and experiences with the medical system differed in their home country from that of those who were born in the U.S., thereby leading to omitted variable bias (Wooldridge, 2010, pp. 89–90). Hence, immigrants are excluded from this study.

## 4 Recent trends in the height of the US-born population

Prior studies have shown that until World War II the U.S. population was the tallest in the world but thereafter their height lagged relative to those observed in the welfare states of Western and Northern Europe, where gains in physical stature accelerated (Fredriks et al., 2000; Komlos & Lauderdale, 2007). By the 1970s birth cohorts, Americans were, on average, 2–6 cm shorter than their counterparts in those countries (Schönbeck et al., 2013; Tanner, 1987a).

**Fig. 2:**
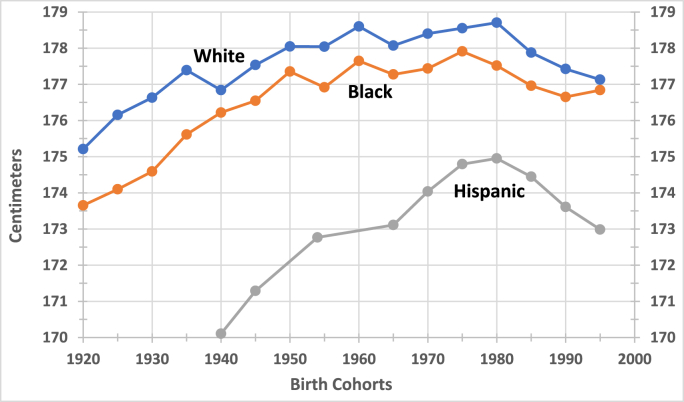
Mean Height of Men Born in the USA Source: Source: See Table 1. Note: The X-axis denotes quinquennial intervals beginning with the year indicated. The 1995 estimate includes the years up to 2003.

**Fig. 3:**
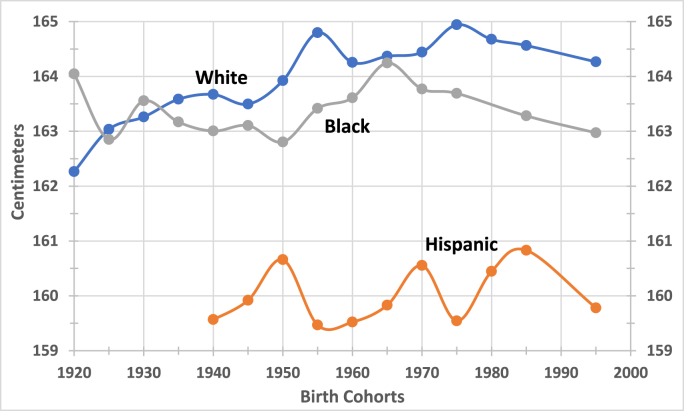
Mean Height of Women Born in the U.S. Source: Source: See Table 1. Note: The X-axis denotes quinquennial intervals beginning with the year indicated. The 1995 estimate includes the years up to 2003.

The principal finding of this study is that even the modest gains in adult height of the postwar era came to a halt by the 1980-84 quinquennium—or earlier—and began to decline across all demographic groups examined, although less convincingly among Hispanic women (Fig. 2, Fig. 3). Hispanic women's height trend shows substantial variability, warranting cautious interpretation of their most recent height decline. Moreover, among black men and white women, the downturn began a quinquennium earlier, while among black women, it appears to have started as early as the late 1960s. Notably, the absolute decline in height of black women was reported earlier (Komlos, 2010). The estimated decline in mean height for men range from 1.10 ± 0.54 cm to 1.97 ± 0.77 cm and for women from 0.68 ± 0.36 to 1.27 ± 0.50 cm and are statistically significant for all six demographic groups, with Hispanic men experiencing the largest decrease and white women the smallest (Table 2). That women's height responds less than that of men to environmental stressors has been documented in multiple studies across diverse populations (Bogin et al., 2017; Giofrè et al., 2025a).

**Table 2:** Decline in height of the U.S.-born population by birth-cohorts.

Men					
	From	Until	cm	st. err.	Signif.

White	1980–84	1995–2003	−1.57	0.56	0.05
Black	1975–79	1985–2003	−1.10	0.54	0.04
Hispanic	1980–84	1995–2003	−1.97	0.77	0.10


Women					

White	1975–79	1990–2003	−0.68	0.36	0.06
Black	1965–69	1990–2003	−1.27	0.50	0.02
Hispanic	1985–89	1990–2003	−1.05	0.54	0.06

## 5 Discussion

The estimated decline in U.S. heights is corroborated by analysis of self-reported height data (Van Dam, 2023), showing a decline of similar magnitude, as well as by the 1.7 cm decline in the mean height of 19-year-old boys and the 0.7 cm decline among 19-year-old girls born between 1981 and 1997 (Fryar et al., 2021)^1^. However, the decline in US heights alluded the NCD Risk Factor Collaborators (NCD Risk Factor Collaboration (NCD-RisC), 2020; Freedman et al., 1997, table 2). Notably, among developed peer countries, U.S. is the only country whose native-born population experienced a diminution in its physical stature (Albertsson-Wikland et al., 2020; Cole & Mori, 2018; Danubio & Sanna, 2008; Holmgren et al., 2019; Lehmann et al., 2017; Rybak et al., 2020; Tinggaard et al., 2014; Schönbeck et al., 2013, Staub et al., 2011; Eiholzer et al., 2025; Kirchengast et al., 2024; Kirchengast et al., 2023; Fudvoye & Parent, 2017; Larnkaer et al., 2006).^2^

Taken together, the nearly simultaneous downturns in two omnibus indicators of biological well-being— average height and the rate of increase in life expectancy—coincided with the onset of profound socio-economic, ideological, and political transformations under the Reagan administration, which sharply increased inequality and precarity. These 'Social-Economic-Political-Emotional' factors--impeded the biological well-being of the population in the long run (Fig. 2, Fig. 3, and Table 2). (Bogin, 2021) After all, the political regime in power establishes the framework within which policies shape population health and longevity (Komlos & Kriwy, 2003; Scheffler et al., 2025).

**Fig. 4:**
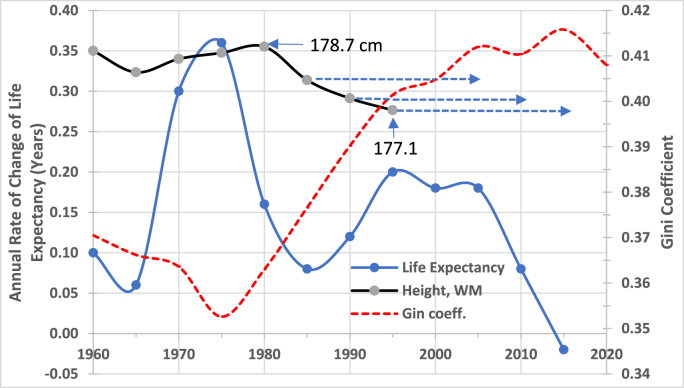
Inequality and the Biological Well-Being of the US population Note: The X-axis denotes quinquennial intervals beginning with the year indicated. The scale for white men's height is the same scale as Fig. 2. The horizontal arrows indicate the two-decade period during which the environmental conditions influence the linear growth of the respective cohorts. Sources: Fig. 1, Fig. 2 and St. Louis Fed series SIPOVGINIUSA.

The *annual* rate of increase in life expectancy, which averaged 0.36 years (4.3 months) during the second half of the 1970s, fell sharply to 0.08 years (29 days) within the following decade (Fig. 4). Nearly simultaneously, the height of white men began to decline—from 178.7 cm among those born between 1980 and 1984 to 177.1 cm among cohorts born between 1995 and 2003.

Note that adult height reflects environmental and socioeconomic conditions experienced during approximately the first two decades of life (plus the prenatal period), as indicated by the horizontal arrows. Over this extended interval—from roughly the mid-1980s through 2023—these cohorts matured amid rising inequality, as reflected by the increase in the Gini coefficient from 0.35 to 0.41. This reversal of the previous egalitarian trend marked the onset of a “stupendous”, “spectacular”, and historically unprecedented rise in inequality, one that had “no equivalent in other rich countries” (Piketty, 2014). The post-tax share of total income accruing to the top 0.1 %, which increased from under 2 percent of total income in 1980 to 7 percent by the end of the century (Piketty & Saez, 2007; Stiglitz, 2012).

In unequal societies, disadvantaged households face reduced access to nutrient-rich foods, prenatal care, and healthcare, compromising maternal nutrition, fetal growth, and ultimately adult stature (Bogin et al., 2017; Reid et al., 2017; Victora et al., 2008). Economic stress and food insecurity, a concomitant of inequality, heighten the risk of stunting, while psychosocial stress elevates cortisol levels, suppressing growth hormone and IGF-1, both essential for skeletal development (German et al., 2020). Over the life course, cumulative stress accelerates aging and increases premature mortality (Case & Deaton, 2020; Johnson et al., 2011; Johnson & Gunnar, 2011; Komlos, 2021a; Oliván, 2003; Terlizzi & Zablotsky, 2024; Zhong et al., 2022). Limited and lower-quality healthcare further exacerbates these disparities, as preventive care, vaccinations, and timely treatment are stratified by income, shaping both childhood growth and adult longevity (Himmelstein et al., 2018; Wilkinson & Pickett, 2009).

Hence, it is notable that the inflection point in the trajectories of both life expectancy and height coincided, in the main, with the onset of the Reagan administration's neoliberal socio-economic policies (Prasad, 2012). Subsequently, public health programs were curtailed as greater responsibility for financing Medicaid was shifted onto the fifty states, while expanded waiver provisions allowed them to reduce the scope of services offered (Rowland et al., 1988). Federal support for neighborhood health programs was also cut, limiting access to preventive and primary care for low-income families (Rothschild, 1982). Moreover, eligibility for unemployment insurance was tightened as states were encouraged to adopt stricter job-search requirements, and funding for Aid to Families with Dependent Children and the food stamp program was reduced (Smithin, 1990). At the same time, the administration promoted an ideology portraying the poor as undeserving while enacting substantial tax cuts for the wealthiest Americans (Pierson, 1994). These changes made it more difficult for subsequent Democratic administrations to reverse course and restore support for vulnerable groups (Abramovitz & Hopkins, 1983).

These policies fostered inequality and insecurity and were compounded by the administration's laissez-faire approach toward both the healthcare and food industries—two crucial determinants of human development—, further undermining public health (Lasch, 1988). In subsequent decades, globalization, technological disruption, the financial crises of 2008, the subsequent recession, and the mainstreaming of right-wing populist movements amplified inequality and social stress, intensifying the adverse impact on population health (Komlos, 2024).

It was challenging that health care costs increased faster than household incomes. For example, between 1984 and 2023, median household income increased by 260 %, while the cost of medical care services increased by 458 %, or by 198 % more. (St. Louis Fed. Fred series CUSR0000SAM2, MEHOINUSA646N) Between 2009 and 2023, health insurance premiums increased by 148 %, compared to just a 62 % increase in median household income (St. Louis Fed. Fred series WPS411103). Consequently, the high cost of insurance left 8.3 % of the population (27 million people) without any health insurance at any time in 2021 and until 2013 the share was closer to 15 % (Keisler-Starkey & Bunch, 2022). Additionally, another 12 % had a gap in coverage at some point during the year, and a further 23 % were underinsured, meaning their insurance “didn't provide them with affordable access to health care”. Many in this group “said that they avoided getting needed health care because of its cost” (Collins & Gupta, 2024). Consequently, only 56 % of the population had adequate insurance coverage throughout the year. Furthermore, approximately 25 % of total healthcare expenditures are estimated to be wasted (Shrank et al., 2019). Thus, Americans pay excessive amounts for health care and receive less health for it than people in other rich countries (Anderson et al., 2019)

The U.S. food environment also contributes to the lagging linear growth, as healthy development depends on the consumption of an adequate combination of nutrients, including essential micronutrients, to support bone and tissue development (NCD Risk Factor Collaboration (NCD-RisC), 2016; Perkins et al., 2016). As Bogin notes, “there are 50 essential nutrients required for growth, maintenance, and repair of the body.” (Bogin, 2001) Deficiencies in amino acids, zinc, vitamin D, or calcium during critical developmental windows can result in stunting (Norris et al., 2022). Prenatal and postnatal nutrition is also vital (Almond & Currie, 2011; Cole, 2000; Karimi et al., 2021).

Like healthcare, the food-provisioning system is structured primarily around free-market principles and is dominated by large multinational fast-food and prepared-food corporations that wield considerable influence over public health policy through lobbying and regulatory capture (Nestle, 2013). Mirroring “Big Pharma's” sway over prescription drug policy, “Big Food” consistently oppose public-interest initiatives such as taxes on sugary beverages, stricter food labelling requirements, and limits on junk-food advertising (Cairns et al., 2009). Additionally, these corporations manipulate consumer preferences through aggressive promotion of hyper-palatable, ultra-processed foods high in fat and sugar but low in essential nutrients (Popkin, 2022; Poti et al., 2015). This profit-driven commercial strategy has fostered an energy-dense diet conducive to sustained weight gain that has played a central role in fuelling the nation's obesity epidemic. The elevated stress experienced by the population has further compounded this trend (Komlos, 2022).

Stress-induced release of cortisol disrupts the regulation of appetite, encouraging emotional eating, especially of calorie-dense “comfort foods” as a coping mechanism (Tomiyama, 2019; Wardle et al., 2011). Elevated cortisol also promotes insulin resistance, which increases the accumulation of visceral fat, raising the risk of obesity and metabolic syndrome (Block et al., 2009; Epel et al., 2000; Kivimäki et al., 2006). In turn, increases in the body mass index (BMI = weight in kg/height in m^2^) during childhood is associated with an earlier, but shorter, adolescent growth spurt (He & Karlberg, 2001). The resulting earlier puberty accelerates closure of the epiphyseal growth plates, thereby limiting healthy linear growth (Ahmed et al., 2009; Freedman et al., 2005; Holmgren et al., 2017, 2021; Popkin et al., 1996; Shalitin & Gat-Yablonski, 2022; Waterlow, 1994). This effect is particularly important in U.S. where 19 % of children and youth are classified as obese (Stierman et al., 2021)^3^.

The exceptionally high prevalence of obesity among black girls and earlier onset of puberty is likely related to the much earlier inflection point in their height trajectory (Table 2) (Kaplowitz et al., 2001). For example, in 1988–1994 the prevalence of overweight or obesity was 9.6 percentage points higher among black girls than among white girls, whereas among boys the pattern was reversed, the prevalence among black boys was 2 percentage points less than among white boys (Troiano et al., 1995, table 2). By 2018, the obesity rate among black women had risen to 56.9 % (Fryar et al., 2020). This pattern may help explain why the decline in height among black women began roughly a decade earlier than that observed for either white women or black man.

Regardless of the precise timing of the downturn, the magnitude of the height declines documented above is notable, given that one study found that a 1 cm decline in male height corresponds to an approximately 0**·**02 point decrease in the Human Development Index (HDI) (Grasgruber et al., 2016). By that estimate, if the average height of U.S. men would not have declined by 1**·**49 cm, the country's HDI would be 0**·**03 points higher, potentially propelling it from the 17th to the 4th place in the global HDI ranking, just behind Switzerland (United Nations Development Programme, 2025, p. 274). Another study found an even stronger association, estimating gains that imply a decline of 0.07 HDI units for men and 0.10 HDI units for women for the average U.S. declines reported in Table 2 (Giofrè et al., 2025b). These estimates are derived from global data—and could overstate the height-HDI relationship for developed nations. However, another analysis, based on U.S. data indicates that the size of the height declines estimated (Table 2) are associated with a 1.5 % reduction in income for men and 0.8 % for women, corresponding to an annual income loss of roughly $1000 for men and $450 for women which might translate into as much as an annual loss of $175 billion at the societal level (Case & Paxson, 2008; St. Louis Fed. Fred series CPIAUCSL, LES1252882800Q, and LES1252881900Q). These findings support the inference that decline in height in the U.S. is consequential for the living standard of the population.

## 6 Conclusion

This study extends existing analysis of height trends among the native-born U.S. population beyond the cohorts examined in prior research. The findings reveal a significant and unique decline in physical stature among individuals born during the final two decades of the twentieth century, and grew up between circa 1980–2023^4^. The downturn coincided with the slowdown in the rate of increase of life-expectancy, together signaling a broader erosion in the nation's biological standard of living during an era that has been characterized by the dominance of neoliberal ideology in its approach to government policy, emphasizing market solutions and individual responsibility, while diminishing the role of the state in addressing issues of public health. Falling behind peer countries in both of these two key demographic and anthropometric indicators with common biological roots, highlights the deep structural shortcomings of U.S. socio-economic policies in promoting healthy human development.

These findings emphasize the value of integrating biological indicators into socioeconomic research and of strengthening policy recommendations addressing the structural determinants of health—nutrition, access to medical care, income equality, and financial security, four pillars of a population's biological well-being. Thus, focusing exclusively on the conventional bellwether indicators of economic performance, such as GDP growth, is insufficient from a humanistic perspective (Komlos, 2021b). The results suggest that the reliance on predominantly market-oriented systems of health care and food provisioning has failed to create conditions conducive to optimal biological development, despite substantial growth of average income and advances in medical technology (Blumenthal et al., 2024; Wolhandler et al., 2021). Reducing inequality, enhancing social protection, and mitigating the psychosocial stress associated with economic precarity are essential for improving the population's biological welfare.

## Ethical statement

Data are publicly available. Ethical considerations do not pertain to this study.

## Funding

This research did not receive any specific grant from funding agencies in the public, commercial, or not-for-profit sectors.

## Declaration of competing interest

John Komlos declares that he has no potential conflicts of interest that may affect the objectivity of this research.

## Data Availability

Data will be made available on request.

## Footnotes

1 The 1 cm decline in height of 19–24-year-olds measured in 1989-1991was statistically insignificant (Freedman et al., 1997).

2 This website reports a decline in heights in the Netherlands: https://longreads.cbs.nl/the-netherlands-in-numbers-2021/how-tall-are-dutch-people/. However, Stef van Buuren, one of the authors of (Schönbeck et al., 2013) confirmed in a personal communication, that the data reported on that website is based on self-reported heights and include immigrants whereas in their study only those born in The Netherlands were included. Moreover, Spanish heights did decline among those born after 1980 (Candela-Martinez et al., 2022). However, this result is unconvincing because the data set includes naturalized immigrants to Spain and in 2024 252,000 people gained citizenship mostly from Morocco, Venezuela and Colombia. Instituto Nacional de Estatdistica, Press Release, June 5, 2025, https://www.ine.es/dyngs/Prensa/en/EANER2024.htm.

3 Obesity among children and youth is defined as being above the 95^th^ percentile of the CDC growth charts (Stierman et al., 2021).

4 The only other period when average heights declined in the U.S. was during the antebellum era, when rising food prices, driven by urbanization and the rapid expansion in the industrial labor force, adversely affected the nutritional status of the population (Komlos, 1998).
